# Skeletal Muscle Atrophy Induced by Dexamethasone Is Attenuated by Amino Acid Complex Supplementation in Rats

**DOI:** 10.3390/life15040517

**Published:** 2025-03-21

**Authors:** So-Jung Lim, Hyun-Jin Kim, Hansik Kim, Heesoo Nam, Kyung-Soo Nam, Inho Kim, Ryun Kang, Inyoung Hwang, Ju-Seop Kang

**Affiliations:** 1 Department of Pharmacology, College of Medicine, Hanyang University, Seoul 04763, Republic of Korea or koreasojung@korea.ac.kr (S.-J.L.);; 2 Department of Physical Education, Graduate School, Korea University, Seoul 02841, Republic of Korea; 3 Bogoshinyak Co., Ltd., the Research Institute, 106, Wonbuk 1-gil, Nonsan-si 32925, Republic of Korea; rnd1@ebogo.kr (H.K.); rnd@ebogo.kr (H.N.); ceo@ebogo.kr (K.-S.N.); 4 The Korean Food Professional Engineers Association, 127 Beobwon-ro, Songpa-gu, Munjeong-dong, Seoul 05386, Republic of Korea; 5 Department of Medical and Digital Engineering, College of Engineering, Hanyang University, Seoul 04736, Republic of Korea; z2021113673@hanyang.ac.kr; 6 Department of Clinical Pharmacology and Therapeutics, Hanyang University Seoul Hospital, Seoul 04736, Republic of Korea

**Keywords:** dexamethasone-induced sarcopenia, treadmill test, forced swimming test, amino acid complex supplementation, M-line, Z-line

## Abstract

Muscle atrophy, a physiological decline in muscle mass and strength due in ageing, occurs through an imbalance between protein breakdown and synthesis. The purpose of this study was to verify whether amino acid complex supplementation (ACS) can prevent and treat muscle loss in a dexamethasone (Dexa, 800 μg/kg)-induced rat model of sarcopenia. Sprague Dawley rats (6 weeks old) were assigned to seven groups: (i) normal control, (ii) positive control (high-dose ACS, 500 mg), (iii) Dexa only, (iv) Dexa + high-dose ACS (500 mg), (v) Dexa + medium-dose ACS (300 mg), (vi) Dexa + low-dose ACS (100 mg), or (vii) Dexa + liquid amino acid complex formulation (LF, 2 mL), administered orally for 4 weeks. Exercise capacity tests were performed five times using a treadmill test (TT) and forced swimming test (FST). The body weight increase in each group was less than that of the normal group. The blood biochemical indices, AST levels, and AST/ALT ratio significantly increased in the Dexa-treated medium-dose ACS group. The total muscle protein also significantly increased in all ACS groups. In the Dexa-treated LF group, CK decreased below the normal level. Exercise capacity, assessed by TT and FST, increased the most in the positive control and Dexa-treated high-dose ACS groups. In the TT, the Dexa-only group increased by about 18%, but the Dexa-treated high-dose ACS group increased by about 110%. Additionally, in the FST, Dexa-treated rats receiving a high dose of ACS demonstrated significantly increased exercise time and capacity. Electron microscopic (EM) and hematoxylin and eosin (H&E) observations of muscle tissue revealed muscle fiber atrophy in the gastrocnemius muscles of the Dexa-only group. In the EM findings of the Dexa-treated high-dose ACS group, the M-line and Z-line were clearer than in the Dexa-only group, and the mitochondria were partially preserved. In conclusion, the ACS-treated rats showed a clear recovery from muscle damage based on serum indices, total muscle protein mass, and the microscopic findings on muscle tissue. Notably, a high dose of ACS demonstrated the most effective protection and recovery of muscle tissue in the Dexa-induced sarcopenia rat model.

## 1. Introduction

Muscle loss caused by decreased activity, malnutrition, inactivity, and hormonal changes can impact the physical health of ageing adults. As life expectancy has increased, the increased medical costs for age-related diseases such as muscular atrophy, osteoporosis, and degenerative neurological diseases have increased social burden. In the elderly population, exercise (activity) depends not only on the neuroregulatory function of the brain, but also on the execution of actions by skeletal muscles. Skeletal muscles is main source of the majority of protein in a healthy human, accounting for approximately 40–50% of total body weight and playing an important role in energy homeostasis. After the age of 30, muscle mass decreases by 3–8% every 10 years, and after the age of 60, muscle mass decreases even more rapidly [1]. Muscle atrophy results in muscle weakness during physical activity. It is usually caused by an imbalance between protein breakdown and synthesis and can be caused by several factors, including muscle wasting, malnutrition, obesity, injury, and cancer-related cachexia [2,3,4].

Sarcopenia is recognized as a multifactorial condition resulting from various medical, behavioral, and environmental influences, commonly observed in older adults [1,4]. Several mechanisms contributing to sarcopenia have been identified, including the degeneration of α-motor neurons in the spinal cord, the impaired secretion of endogenous growth hormone and insulin-like growth factor-1 (IGF-1), androgen and estrogen deficiencies, insufficient dietary protein intake, the increased expression of catabolic cytokines, and decreased physical activity [5,6]. It is widely accepted that age-related alterations in body composition are influenced by reductions in anabolic hormone levels, neuro-muscular modifications, and a decline in muscle protein turnover [7].

These deficits in both the quantity and quality of contractile proteins contribute to physical disability, frailty, loss of functional independence, increased risk of falls and fractures, and higher healthcare expenditures [8,9]. Consequently, the decline in muscle mass and prolonged physical inactivity in elderly individuals may reduce insulin sensitivity, thereby impairing glucose uptake, storage, and utilization in peripheral tissues, particularly skeletal muscle. Within this context, the dietary requirements for amino acids (AA) and protein may be significantly elevated in elderly individuals to counteract muscle catabolism and promote protein synthesis [10,11].

Restoring anabolic conditions that promote endogenous protein synthesis and cellular adenosine triphosphate (ATP) production may have beneficial effects on muscle integrity and metabolic function, ultimately enhancing insulin activity and sensitivity. This approach may be particularly advantageous for elderly individuals with sarcopenia and could be effectively achieved through AA supplementation [12,13,14]. Therefore, regulating the key factors involved in protein breakdown and synthesis is important for preventing muscle wasting [15].

Increased protein degradation through the ubiquitin–proteasome system and autophagy stimulates muscle protein catabolism, leading to muscle wasting [3]. The rate of muscle protein breakdown is proportional to the serum creatine kinase (CK) (muscle type) level, an enzyme used as a marker for muscle protein breakdown [5]. Decreased Akt phosphorylation stimulates the Forkhead box protein O1 (FOXO1) transcription factor, which induces Atrogin-1 and muscle RING-finger protein-1 (MuRF1), ubiquitin ligase proteins that are key hallmarks of muscle atrophy signaling. In addition, muscle protein anabolism is regulated by Akt phosphorylation, and decreased Akt phosphorylation inhibits protein synthesis via the mTOR/p70S6K pathway in skeletal muscle [3,6]. Dexamethasone (Dexa), a synthetic steroid, has been widely used to establish animal models of muscle atrophy [3,16,17]. In mouse animal models, intraperitoneal injections of Dexa for 5 days induce muscle damage. The Dexa-induced atrophy is associated with a disruption of the balance between protein degradation and synthesis due to decreased Akt phosphorylation, leading to muscle wasting [7,8].

Amino acids are potent stimulants of muscle protein synthesis in both young and elderly persons [9,10]. However, the anabolic response to a mixed meal containing both amino acids and carbohydrates is reduced in elderly persons [18]. Therefore, the loss of muscle mass and strength that occurs with ageing may be partly due to suboptimal protein intake. A nutritional supplement containing amino acids may be a practical approach to improving muscle mass and strength in elderly persons. Previous studies in elderly persons have shown that dietary supplementation is not effective in improving lean body mass [11,13,14]. However, it is necessary to consume an equivalent number of calories per day from supplements, as nutritionally balanced supplements have been shown to reduce caloric intake from the rest of the diet [13]. Therefore, in the case of older individuals, dietary supplements may be considered a more appropriate dietary replacement.

Consequently, it is best to provide the most nutritionally effective supplement possible. From this perspective, it was determined that an intake of essential amino acids (EAAs) is necessary to stimulate muscle protein synthesis [19]. Furthermore, the stimulatory effect of EAA on muscle protein synthesis is more than twice that of the same amount of high-quality protein [20]. In particular, arginine is believed to possess unique acute anabolic effects [21].

The purpose of the present study was to verify whether amino acid complex supplementation (ACS) is effective in preventing and treating muscle loss in Dexa-induced sarcopenia in rats, and to attempt to develop amino acid supplements for the prevention and treatment of sarcopenia in elderly persons with various causes in the future.

## 2. Subjects and Methods

### 2.1. In Vitro Experiments: Cytotoxicity of ACS by MTT Method

Cell viability was measured by assessing the degree of color change induced by EZ-CYTOX (DoGen, Seoul, Republic of Korea), a reagent that reacts with dehydrogenase present in mitochondria (intracellular organelles). This was achieved by serially diluting samples of high-concentration ACS (Bogoshinyak Co., Chungcheongnam-do, Republic of Korea) Pharm in a hepatocyte cell line and culturing them over a certain period. The cytotoxicity pattern was confirmed based on the MTT test results [22].

### 2.2. In Vivo Experiments

**Figure 1:**
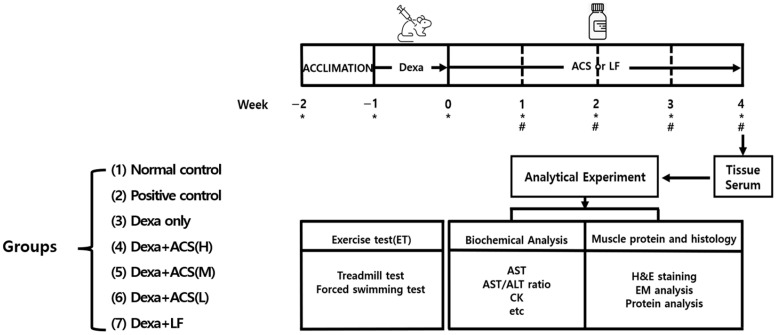
Experimental protocol, groups, and analytical procedures. Dexa: dexamethasone (800 μg/kg per day) for 5 consecutive days; ACS and LF (oral dose daily for 4 weeks at a set dosage); # exercise test (one time per week). * body weight (per week for 7 weeks).

Male Sprague Dawley rats (SR strain, 6 weeks old) were purchased from Orient Bio Inc. (Seongnam, Republic of Korea). Dexa was purchased from Sigma-Aldrich (St. Louis, MO, USA). The muscle atrophy animal model was established by modifying a previously developed protocol [23]. The rats were maintained in an SPF room at a temperature of 23 ± 2 °C, a humidity of 60 ± 5%, and a 12 h light/dark cycle. The rats had free access to drinking water and were fed a standard laboratory chow diet (LabDiet 5001, Land O’Lakes, Inc, Arden Hills, MN, USA). The experimental group assignment and experimental protocol were as follows: After an adaptation period, the rats were randomly assigned to 7 groups *(n* = 5) based on initial body weight: (i) Normal control; (ii) Positive control with high-dose ACS (500 mg); (iii) Dexa only (800 μg/kg, ip); (iv) Dexa+high-dose ACS (H, 500 mg); (v) Dexa+medium-dose ACS (M, 300 mg); (vi) Dexa+low-dose ACS (L, 100 mg); and (vii) Dexa+liquid amino acid complex formulation (LF, 2 mL). The rats were orally administered the ACS solution (500, 300, or 100 mg, po) for 4 weeks; the solution treatment (2 mL) was administered orally, and saline was administered orally to the rats in the control and Dexa only groups (Figure 1). The dose of ACS used in this animal study was based on doses used in previously reported Dexa-induced muscle atrophy [3,16,17] and the clinical dose suggested by the manufacturer. The dose of ACS used in the 4-week animal study on muscle atrophy was based on the dose administered to humans suggested by the manufacturer. The Dexa only group received intraperitoneal injections of Dexa (800 µg/kg, ip) for 5 consecutive days. The Normal control and the ACS-alone groups received intraperitoneal injections of saline. During the 4-week animal study, the body weight of each rat was measured weekly before supplementation with the ACS and Dexa administration. The exercise capacity test was performed three times according to the protocol, using treadmill and forced swimming methods. The length (seconds) of continuous exercise without becoming tired was measured. At the end of the experimental period, the animals were sacrificed according to the protocol. Whole blood was collected to measure liver function and muscle indices. Additionally, muscles (gastrocnemius and soleus) were collected to observe the histological examination of muscle protein content, and muscle tissue morphology was assessed using a light microscope (H&E, hematoxylin and eosin double stain) and an electron microscope (EM). The experiments performed in this study were approved by the Animal Experiment Management Committee of Hanyang University (IACUC approval number (HY-IACUC-25-0113; 25 April 2024).

### 2.3. Statistical Analysis

All data are expressed as mean ± SD (*n* = 5). Statistical analyses and analysis of variance with one-way ANOVA, followed by Tukey’s post hoc multiple comparison test, were performed using IBM SPSS version 24 (Systat, Chicago, IL, USA), and *p* < 0.05 was considered statistically significant.

## 3. Results

### 3.1. In Vitro Test: MTT Test (Cytotoxicity of Amino Acid Complex)

**Figure 2:**
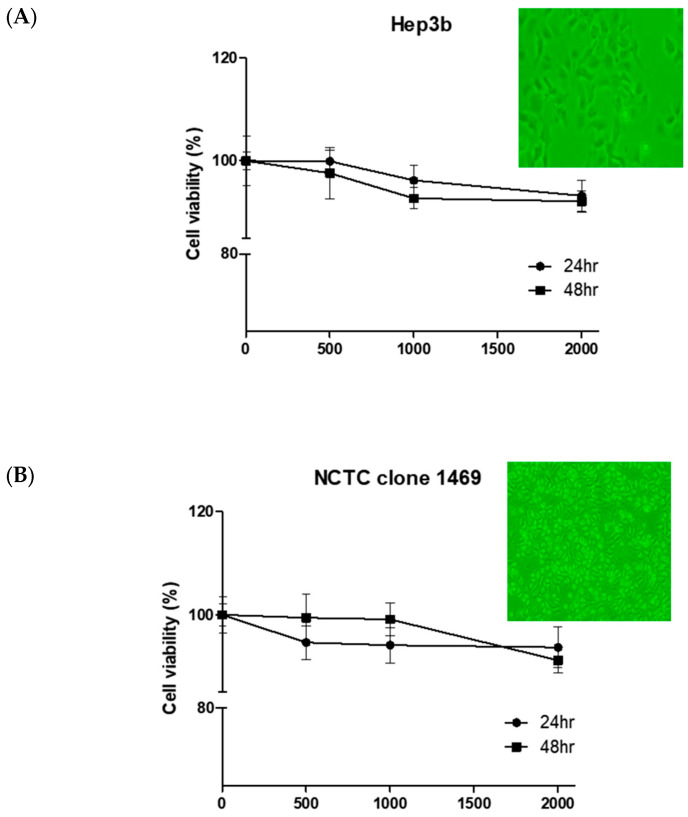
Cell viability after treatment with the amino acid complex. Cell viability was maintained at over 90% in (**A**) the hepatocyte cell line (NCTC 1496) and (**B**) in liver cancer cells (Hep3b) for up to 48 h after treatment with up to 2 μg.

As shown in Figure 2, the cell viability was maintained at over 90% for all ACS concentrations (0, 500, 1000, and 2000 ng/mL) after 48 h of culture, indicating that no cytotoxicity was observed in either normal hepatocytes or liver cancer cell lines within the range of possible administration doses (Figure 2).

### 3.2. In Vivo Experiment: Body Weight

**Figure 3:**
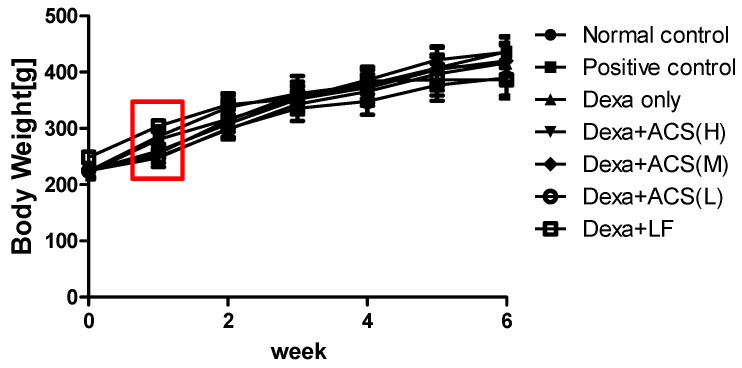
Body weight change. The red square indicates significant weight loss after the administration of dexamethasone.

Dexa was administered at a predetermined dose for 1 week and measured weekly. Significant weight loss was observed compared to the control group, indicating that sarcopenia was induced successfully. The average body weight after Dexa administration was 258.6 ± 9.6 g, and the normal control group was 292.9 ± 11.5 g, indicating that sufficient muscle loss occurred due to Dexa. No changes in food intake or appearance were observed during this period in all groups. During the experiment period, the weight in each group tended to increase less than that of the normal control group, but there was no statistical significance (Figure 3).

### 3.3. In Vivo Experiment: Changes in Blood Biochemistry

**Figure 4:**
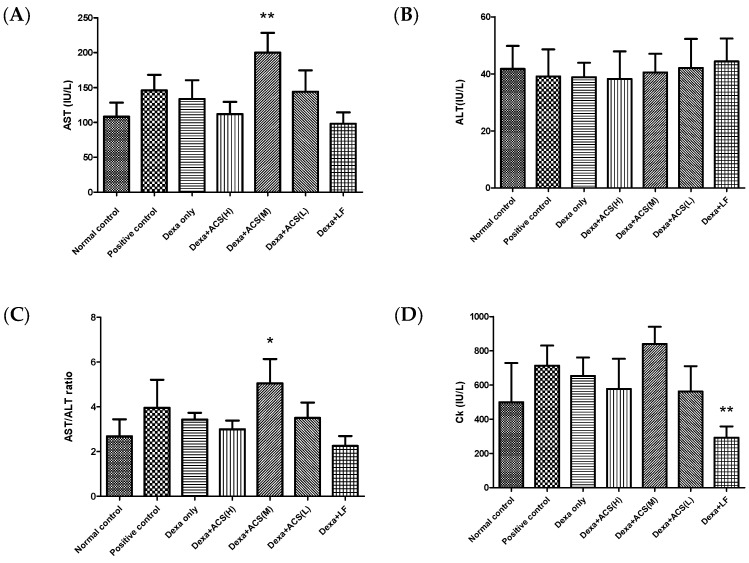
Effects of ACS on (**A**) AST, (**B**) ALT, (**C**) AST/ALT ratio, and (**D**) CK levels in the Dexa-induced sarcopenia in rat. The data represent the mean ± SD of 5 rats. The symbols in the figures show statistically significant differences among the groups as follows: * *p* < 0.05, ** *p* < 0.001 vs. the Dexa only group.

To evaluate the effect of the amino acid complex on blood chemistry, changes in blood biochemical indices were measured. As shown in Table 1 and Figure 4, in the Dexa+ACS (M) group, the level of AST and the AST/ALT ratio significantly increased, but ALT did not change significantly. Serum CK significantly decreased in the Dexa+ACS group and was similar to the normal control group. However, there was no difference between the groups in the other indices. Although there was no dose dependency, levels of AST and the AST/ALT ratio significantly increased in the medium-dose group. Therefore, it was determined that ACS had some effect on liver cells. This may be related to muscle damage because exercise power recovery was not complete at medium doses.

ALT is highly specific for liver toxicity, and AST indicates broad toxicity to liver cells, muscle cells, etc. However, since ALT is not high and AST and CK are high, this may be an effect derived from muscle damage rather than from the liver. It may be related to muscle damage because exercise power recovery was not complete at medium doses.

In addition, CK, one of the muscle damage indices, fell below the normal level in the Dexa+ACS group, so it was determined that it had a beneficial effect on muscular damage in Dexa-induced sarcopenia.

### 3.4. In Vivo Experiment: Exercise Capacity Test

**Figure 5:**
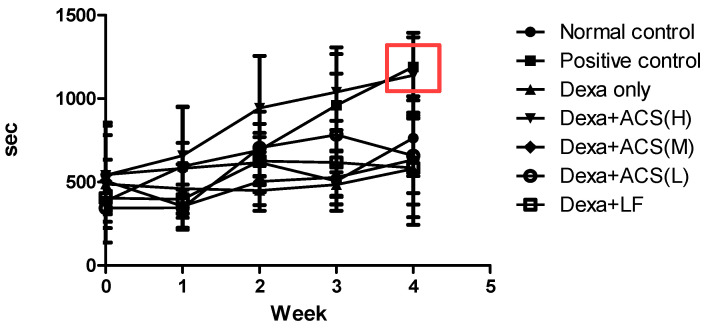
Changes in exercise volume and exercise capacity on the treadmill test. The red square indicates the greatest improvement in the positive control and Dexa+ACS (H) groups after ACS supplementation.

In the treadmill test, the Dexa control group exhibited an 18% increase over four weeks, whereas the Dexa+ACS (H) group showed a significantly greater increase of approximately 110%. The Dexa only group presented a result of 485 ± 61 s, but the high-dose group showed a significant increase to 946 ± 89 s, and the positive control (989 ± 101 s) showed a similar increase. The normal control group demonstrated a similar improvement to the Dexa+ACS (H) group. The other groups showed a slight increase of 500~700 s, but there was no statistical significance (Figure 5).

**Figure 6:**
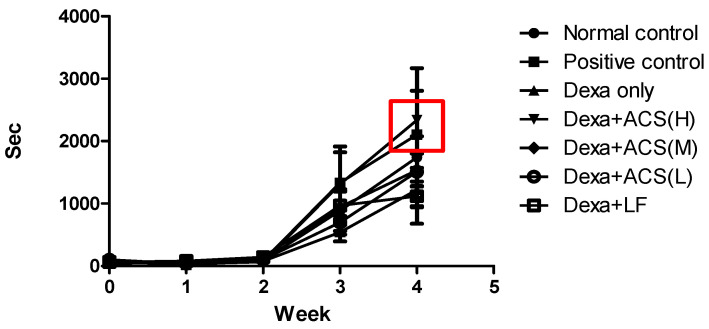
Changes in exercise volume and exercise capacity in the forced swimming test. The red square indicates the greatest improvement in the positive control and Dexa+ACS (H) groups after amino acid complex supplementation (ACS).

In the forced swimming test, both exercise amount and exercise capacity significantly increased in the Dexa+ACS (H) group. The Dexa only group had 734 ± 67 s, but the high-dose group showed a significant increase to 1452 ± 130 s, and the positive control (1166 ± 105 s) showed a similar increase. The other groups showed a slight change of 660~909 s, but there was no statistical significance (Figure 6).

Overall, the Dexa+ACS (H) group displayed comparable improvements in exercise volume and performance across both the treadmill and forced swimming tests (Figure 5 and Figure 6).

### 3.5. In Vivo Experiment: Total Amount of Protein in Muscle Tissue

**Figure 7:**
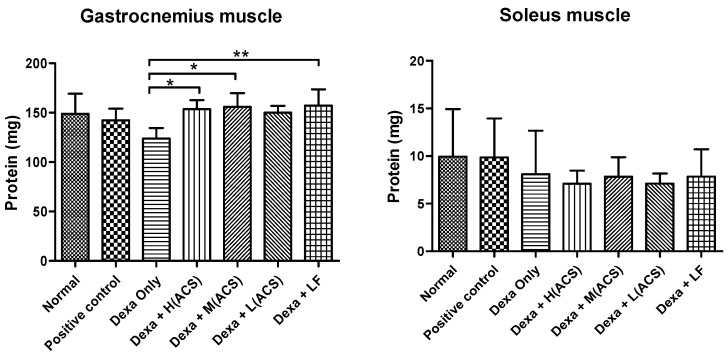
Changes in total protein content in the gastrocnemius and soleus muscle. * *p* < 0.05, ** *p* < 0.001 vs. the Dexa only group.

The measurement of the total muscle protein in the gastrocnemius and soleus muscles revealed that the change in the gastrocnemius was significant, but the change in the soleus muscle was not significant. In the gastrocnemius muscle of each group, the Dexa only showed a significant decrease compared to the normal control. However, in the Dexa+ACS and Dexa+LF groups, the trend in muscle protein recovery was significant (Figure 7).

### 3.6. In Vivo Experiment: Histological Observation of Liver and Muscle Tissues

**Figure 8:**
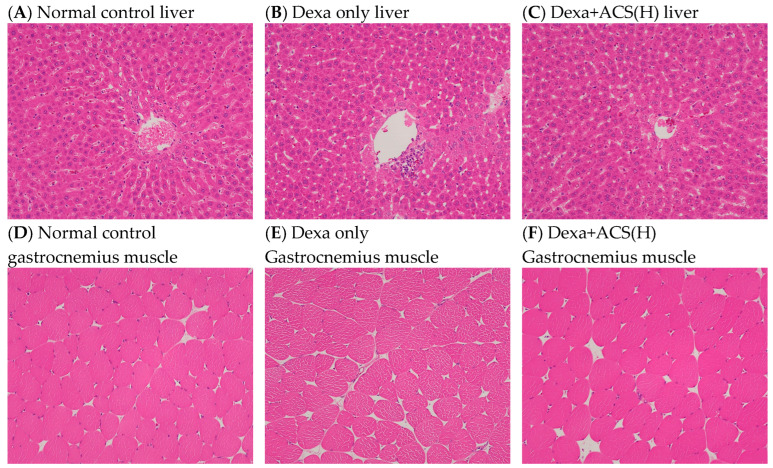
H&E staining [liver and gastrocnemius muscle, ×100].

**Figure 9:**
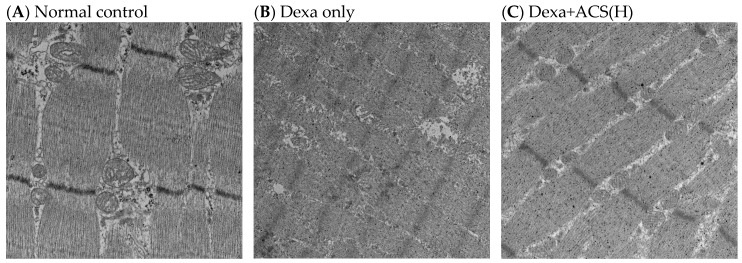
Electron microscopic findings [gastrocnemius muscle, ×5000].

For each group, liver and muscle tissue were collected from two experimental animals that completed the experimental procedure, and histological observations were performed using H&E staining and electron microscopy. The effects of the experimental substances on the liver tissue were observed using H&E staining, and the inflammation response (inflammation, score 0–3), hepatocyte damage (hepatocyte degeneration/necrosis, score 0–4), and fat deposition (steatosis, score 0–3) of the liver tissue were assessed according to a validated evaluation method [24,25]. Unlike the normal control group, the positive control group showed one point of hepatocyte damage and the Dexa only group showed one point of inflammation and one point of hepatocyte damage. However, similar to the normal control group, the Dexa+CS (H) group showed no damage. The Dexa+ACS (M) group showed 1 point of inflammation and 0.5 points of hepatocyte damage and the Dexa+ACS (L) group and Dexa+LF group showed 0.5 points of inflammation. Therefore, no abnormal findings were observed in the liver tissue of the Dexa-induced sarcopenia rat model when a high dose of ACS was administered, similar to the normal control group (Figure 8). Only the normal group, the Dexa control group, and the Dexa+ACS (H) group were examined by EM. In the muscle cell findings of the normal control group, the representative M-lines and Z-lines of muscle fibers were clearly shown, and the mitochondria of the muscle fibers were also preserved intact. Conversely, in the muscle cell findings of the Dexa only group, the M-lines and Z-lines were unclear or disconnected, and the mitochondria also appeared to be largely destroyed. However, in the muscle cells of the Dexa + ACS (H) rats, the M-lines and Z-lines were maintained more clearly than in the Dexa-only group, and the mitochondria were partially preserved and intact. Based on these findings, it seemed that some of the muscle damage caused by dexamethasone was reversed (Figure 9).

## 4. Discussion

Sarcopenia describes the progressive loss of muscle mass associated with immobility or ageing, leading to muscle weakness, prolonged inactivity, and increased susceptibility to injury. The underlying mechanisms of sarcopenia remain unclear. However, an imbalance between protein synthesis and degradation contributes to muscle protein loss [26]. Therefore, sarcopenia results from the progressive loss of skeletal muscle and is associated with diminished physical function, increased risk of falls and fractures, disability, hospitalization, and reduced quality of life [27]. Moreover, sarcopenia is associated with an increased mortality risk in the elderly, with rates rising by up to 2.34-fold [28]. Although exercise enhances muscle mass and strength [29], older adults, particularly those who are physically frail, often face challenges in maintaining regular physical activity. Dietary and nutritional interventions have been shown to support muscle mass and preserve physical performance and function [30]. Emerging evidence suggests that protein supplementation may aid in sarcopenia prevention [31,32,33]. Proteins, composed of amino acids, stimulate muscle protein anabolism, a process influenced by the availability of branched-chain amino acids (BCAA) such as leucine, isoleucine, and valine [34]. AAs can antagonize muscle catabolism, improving global protein synthesis in skeletal and cardiac muscle [10,18,35]. Nutritional supplements with an oral AA mixture significantly increased whole-body lean mass in elderly subjects with sarcopenia. The improvement in the amount of whole-body lean mass could be linked to increased insulin sensitivity and anabolic conditions related to IGF-1 availability [12]. Ageing-induced muscle adaptations can be partly restored by AA supplementation in the aged rat model [36].

However, ageing is associated with delayed amino acid absorption and anabolic resistance, which may impair muscle protein synthesis [37]. Previous studies suggest that individual amino acid supplementation may have detrimental health effects. However, specific combinations of amino acids in dietary supplements have been shown to promote muscle mass and strength gains in sarcopenic elderly individuals, potentially reducing the risk of type 2 diabetes mellitus (T2DM) while mitigating sarcopenic effects [38].

The current study provides experimental evidence that ACS alleviates Dexa-induced sarcopenia via the recovery of muscle cell damage and also prevents declines in exercise capacity in a sarcopenia rat model. The improved exercise capacity in the Dexa-induced muscle damage animals receiving ACS may have been linked to increased muscle protein synthesis, which minimizes the extent of muscle atrophy resulting from Dexa. The beneficial effects of ACS in the Dexa-induced muscle atrophy model should be clarified in future studies of muscle wasting in clinical conditions that may be induced by the adverse effects of various drugs or immobility-induced sarcopenia in older individuals.

Under the given conditions in this experiment, hepatocytes and liver cancer cells were cultured without any complications. In the cytotoxicity (MTT test) results, cell viability was maintained at over 90% for all amino acid complex contents (0, 500, 1000, and 2000 ng/mL) after 48 h of culture. This indicates that no cytotoxicity was observed in normal hepatocytes and liver cancer cell lines within the range of possible administration doses. During the experimental protocol, measurements were recorded once a week and Dexa was administered at a fixed dose for one week. Consequently, sarcopenia was induced with weight loss that showed a significant difference from the normal control group [20]. During the experimental period, the weight change in each group tended to increase less than that of the normal control group, but there was no statistical significance. In the blood biochemical indices measured to evaluate the effect of ACS, AST significantly increased in the Dexa+ACS (M) group, but there was no significant change in ALT of the Dexa only group. In addition, the AST/ALT ratio significantly increased in the Dexa+ACS (M) group. Serum CK significantly decreased in the Dexa+LF group and was similar to the normal control group. Although there was no dose dependence, levels of AST and the AST/ALT ratio significantly increased at the ACS (M) dose, so it was judged to affect muscle rather than liver cells to some extent. Additionally, CK, one of the muscle damage indicators, decreased below normal levels in the Dexa+LF group, so it was judged to have a beneficial effect on Dexa-induced sarcopenia.

In the treadmill and forced swimming tests, which measured exercise capacity on an animal treadmill and in a water tank once a week, exercise capacity improved in the Positive control and Dexa+ACS (H) groups after 4 weeks of administration. In the treadmill test, the capacity of the Dexa only group increased by about 18% over 4 weeks, but the capacity of the Dexa+ACS (H) group increased by about 110%. The positive control group also showed a similar increase. In the forced swimming test, as in the treadmill test, the exercise capacity significantly increased in the Dexa+ACS (H) group. Measurements of the total muscle protein of the gastrocnemius and soleus muscles revealed that the change in the gastrocnemius was significant, but the soleus muscle did not show a significant change. In the gastrocnemius, the Dexa only group showed a significant decrease compared to the normal control group, but the Dexa+ACS group showed a significant increase regardless of the administration dose, indicating a clear tendency for recovery from muscle protein damage.

Liver and muscle tissues were collected from two of the experimental animals following the experimental procedure, and histological observations were performed using H&E staining and EM. The effects of the experimental substance on the liver tissue were observed using H&E staining, and the inflammation response (inflammation, score 0–3), hepatocyte damage (hepatocyte degeneration/necrosis, score 0–4), and fat deposition (steatosis, score 0–3) of the liver tissue were evaluated according to a validated evaluation method. Unlike the normal group, the positive control group showed one point of liver cell damage and the Dexa only group showed one point of inflammation and one point of liver cell damage. However, similar to the normal group, the Dexa+ACS (H) group showed no abnormal findings. The Dexa+ACS (M) group showed 1 point of inflammation and 0.5 points of liver cell damage and the Dexa+ACS (L) and Dexa+LF groups showed the same 0.5 points of inflammation. As with the normal control, no abnormal findings were observed in the liver tissue of the Dexa+ACS group when a high dose was administered. In the muscle tissue, the H&E findings of the gastrocnemius muscle showed some muscle fiber atrophy in the Dexa only group, and no other lesions were observed. Only the normal control, Dexa only, and Dexa+ACS (H) groups were examined with EM. In the muscle tissue and cells of the normal control group, the representative M-line and Z-line of the muscle fiber were clearly visible, and the mitochondria of the muscle fiber were also intact. However, in the Dexa only group, the M-line and Z-line were unclear or disconnected, and the mitochondria were severely destroyed. In the Dexa+ACS (H) group, the M-line and Z-line were more distinct than in the Dexa only group, and the mitochondria were partially preserved and intact. Based on these findings, it seemed that some muscle damage caused by dexamethasone was recovered.

## 5. Conclusions

The MTT assay confirmed that the amino acid complex is a biosafe substance, exhibiting no cytotoxicity within the administrable dosage range. Serum indices showed a beneficial effect in the Dexa+ACS (H) and Dexa+LF groups. Additionally, the Dexa+ACS (H) group showed a significant increase in exercise capacity in both the treadmill and forced swimming tests. Muscle total protein analysis indicated that ACS administration effectively mitigated Dexa-induced sarcopenia, irrespective of dosage. No abnormal findings were observed in the river tissue examination of the Dexa+ACS (H) group. Additionally, histological analysis, including H&E staining and EM, showed a distinct recovery effect in the muscle tissue of the ACS-treated groups. These findings suggest that high-dose ACS exerts the most substantial beneficial effect on muscle tissue. Our study demonstrates that nutritional supplementation with an oral ACS complex may have beneficial effects on muscle function and muscle restoration in a dexamethasone-induced sarcopenia rat model. Further clinical studies are needed to evaluate the effects of oral ACS complex supplementation on muscle loss prevention and exercise capacity in older adults.

## Figures and Tables

**Table 1:** Serum biochemistry.

	Groups *(n* = 5)	Normal Control	Positive Control	Dexa Only	ACS Groups			Dexa + LF
Parameters					Dexa+ACS (H)	Dexa+ACS (M)	Dexa+ACS(L)	
AST (IU/L)		108.3 ± 20.07	146.0 ± 22.12	133.64 ± 26.93	111.80 ± 17.74	200.04 ± 28.44 **	144.02 ± 30.64	98.12 ± 16.22
ALT (IU/L)		41.72 ± 8.05	39.06 ± 9.52	38.78 ± 5.10	38.18 ± 9.67	40.44 ± 6.60	42.04 ± 10.20	44.36 ± 8.00
AST/ALT		2.68 ± 0.76	3.96 ± 1.26	3.43 ± 0.30	2.99 ± 0.39	5.05 ± 1.09 *	3.50 ± 0.69	2.25 ± 0.44
ALP (IU/L)		589.56 ± 266.3	574.00 ± 227.69	572.88 ± 236.27	645.46 ± 222.78	721.46 ± 350.87	563.00 ± 130.91	599.08 ± 04.98
LDH (IU/L)		741.18 ± 464.0	702.70 ± 263.06	670.34 ± 232.76	857.88 ± 317.14	575.18 ± 70.99	492.50 ± 216.13	356.80 ± 38.54
CK (IU/L)		499.8 ± 228.08	712.92 ± 118.24	652.98 ± 107.39	576.98 ± 176.22	839.42 ± 100.69	561.72 ± 148.17	291.70 ± 66.00 **
GLU (mg/dL)		197.30 ± 56.34	161.08 ± 33.33	148.16 ± 36.18	177.36 ± 22.34	150.04 ± 37.07	198.70 ± 166.83	184.40 ± 29.71
T-Chol (mg/dL)		59.24 ± 14.08	56.82 ± 15.93	59.88 ± 11.16	61.94 ± 8.81	50.62 ± 11.09	57.30 ± 9.96	63.74 ± 7.58
TG (mg/dL)		53.48 ± 35.31	55.80 ± 13.96	48.70 ± 14.14	60.16 ± 14.09	64.44 ± 38.65	70.44 ± 41.68	60.68 ± 15.10
T-Pro (g/dL)		6.41 ± 0.61	6.25 ± 0.75	6.63 ± 0.39	6.46 ± 0.20	6.48 ± 0.43	6.79 ± 0.52	6.79 ± 0.29
Alb (g/dL)		2.58 ± 0.28	2.66 ± 0.24	2.75 ± 0.12	2.67 ± 0.18	2.70 ± 0.15	2.72 ± 0.31	2.77 ± 0.10
T-Bil (mg/dL)		0.14 ± 0.02	0.14 ± 0.01	0.15 ± 0.02	0.15 ± 0.01	0.15 ± 0.01	0.14 ± 0.02	0.15 ± 0.01

Data represent the mean ± SD of 5 rats. * *p* < 0.05, ** *p* < 0.001 (significant difference between the other groups and the Positive control). One-way ANOVA, followed by the Tukey’s multiple comparison test.

## Data Availability

Data are contained within the article.
